# Evolutionary origin and asymmetric subgenomic retention of the lncRNA *pGhFAD2–1* that regulates cotton lipid metabolism

**DOI:** 10.3389/fpls.2026.1841757

**Published:** 2026-05-08

**Authors:** Haihong Chen, Xuan Liu, Ni Yang, Zhaolong Gong, Fenglei Sun, Shiwei Geng, Juyun Zheng, Shuaishuai Qian, Junduo Wang, Yajun Liang

**Affiliations:** 1 National Cotton Engineering Technology Research Center, Cotton Research Institute of Xinjiang Uyghur Autonomous Region Academy of Agricultural Sciences, Wulumuqi, Xinjiang, China; 2 Agricultural Genomics Institute at Shenzhen, Chinese Academy of Agricultural Sciences, Shenzhen, China

**Keywords:** cottonseed oil, evolutionary dynamics, *Gossypium*, pGhFAD2-1, structural variation

## Abstract

The origin and evolutionary dynamics of long non-coding RNAs (lncRNAs) represent a pivotal frontier in plant functional genomics. Here, we investigated *pseudo-GhFAD2-1* (*pGhFAD2-1*), a functionally validated lncRNA regulating cottonseed fatty acid metabolism in *Gossypium hirsutum*, delineating its evolutionary origin and structural dynamics through a comprehensive cross-genomic comparative strategy. Homologous sequence identification, phylogenetic reconstruction, and molecular evolutionary analyses were conducted across 27 *Gossypium* species representing the A, B, D, E, F, G, K, and AD genomes. Our findings reveal that *pGhFAD2–1* originated from a tandem duplication event of *GhFAD2-1D* in the D-genome ancestor approximately 5 million years ago (MYA). This duplicated locus was subsequently disrupted by a 1,221-bp exogenous sequence insertion, which abolished its protein-coding capacity and catalyzed the emergence of this novel lncRNA transcript. Subjected to strong purifying selection, this reorganized locus retained its core functional domain and exhibited high-fidelity inheritance (sequence identity>85%) across D-genome diploids and AD-genome allotetraploids. Conversely, only fragmented remnants or complete deletions of this locus were detected in non-D genome *Gossypium* species. This study systematically characterizes the evolutionary trajectory and subgenomic conservation of *pGhFAD2-1*, elucidating its adaptive significance as a D-lineage-specific lncRNA. These findings provide novel insights into how lineage-specific structural variations rewire epigenetic regulatory networks to drive adaptive diversification in allopolyploid crops, establishing an evolution-informed molecular target for the synergistic genetic improvement of cotton fiber and seed oil traits.

## 1 Introduction

Lineage-specific evolution of long non-coding RNAs (lncRNAs) and the subsequent rewiring of epigenetic regulatory networks are crucial drivers of adaptive phenotypic diversification in polyploid plants. In upland cotton (*Gossypium hirsutum* L), a premier dual-purpose crop for natural fiber and edible oil, optimizing cottonseed lipid metabolism—particularly the oleic acid (C18:1) to linoleic acid (C18:2) ratio—has become a primary breeding objective to improve oil oxidative stability and nutritional value (Wu et al., 2022). The conversion of C18:1 to C18:2 in cotton is predominantly catalyzed by the microsomal ^Δ12^fatty acid desaturase 2 (*FAD2*) family (Liu et al., 2002; Ma et al., 2021). In higher plants, the *FAD2* gene family represents a classic model of evolutionary subfunctionalization. Comparative genomic studies across diverse taxa, including Arabidopsis (Okuley et al., 1994), soybean (Heppard et al., 1996; Schlueter et al., 2007; Lakhssassi et al., 2017), and peanut (Chi et al., 2011), underscore that *FAD2* genes frequently originate through tandem and whole-genome duplications, subsequently undergoing functional divergence to satisfy distinct physiological requirements (Dar et al., 2017). Rather than conforming to rigid tissue-specific categories, these duplicates typically exhibit a continuous spectrum of spatiotemporal expression profiles. Through this evolutionary partitioning, specific members maintain basal transcript abundance throughout vegetative organs such as roots, stems, and leaves to ensure cellular membrane homeostasis, while others are preferentially recruited to drive the specialized accumulation of storage lipids during seed maturation (Heppard et al., 1996; Dar et al., 2017). Recent genomic analyses have revealed that the *GhFAD2* gene family has significantly expanded in allotetraploid cotton, comprising nine homologous members distributed across the At and Dt subgenomes (Paterson et al., 2012). While these duplicated members exhibit broad functional divergence across various tissues, the *GhFAD2–1* orthologous pair (comprising *GhFAD2-1A* and *GhFAD2-1D*) acts as the master regulator during cottonseed development (Liu et al., 2019; Ma et al., 2021).

Notably, an additional unique locus—originally annotated as the protein-coding gene *Gh_D13G2237*—is located tandemly adjacent to *GhFAD2-1D* on chromosome D13. Our recent functional characterization demonstrated that this locus lacks the conserved histidine boxes essential for typical *FAD2* enzymatic activity. Instead, it functions as a lineage-specific lncRNA, subsequently designated as *pseudo-GhFAD2-1* (*pGhFAD2-1*), which epigenetically suppresses cottonseed lipid accumulation by recruiting the histone deacetylase GhHDT1 to remodel the local chromatin landscape (Chen et al., 2025). Despite its well-documented biological function in lipid metabolism, the evolutionary trajectory and the genomic rearrangements underlying the genesis of *pGhFAD2–1* across the *Gossypium* phylogeny remain largely elusive (Huang et al., 2021). Elucidating this phylogenetic trajectory is essential to determine whether it represents an ancestral genomic feature shared among early *Gossypium* lineages or a recent innovation arising from subgenome-specific structural variations (Glaszmann et al., 2010). Understanding this origin provides broader insights into the *de novo* birth of regulatory lncRNAs and the genetic architecture underlying adaptive trait divergence in polyploid crops (Wu et al., 2017).

To address this, we conducted a comprehensive phylogenomic survey across 27 representative *Gossypium* species, encompassing the major diploid genomes (A, B, D, E, F, G, and K) and the allotetraploid genomes (AD). By integrating fine-scale structural variation analyses, population evolutionary dynamics, and macroscopic phenotypic evaluations, we aimed to delineate the precise *de novo* origination trajectory of this lineage-specific lncRNA (Li et al., 2023). Our findings illustrate how structural variations drive the “duplication-insertion-neofunctionalization” of a pseudogenized duplicate, providing insights into the evolutionary trade-offs shaping cottonseed lipid metabolism and offering an evolution-based molecular target for synergistic crop improvement.

## 2 Materials and methods

### 2.1 Genomic data retrieval of *Gossypium* species

The reference genome assemblies and annotation files of 27 *Gossypium* species representing diverse evolutionary clades were retrieved from the CottonGen database (https://www.cottongen.org) and the NCBI GenBank database (https://www.ncbi.nlm.nih.gov/genbank/). These datasets included *Gossypium hirsutum* L. acc. TM-1 (AD_1_), *Gossypium barbadense* L. acc. Hai7124 (AD_2_), *Gossypium herbaceum* L. acc. Zhongcao 1 (A_1_), *Gossypium arboreum* L. acc. Zhongya 1 (A_2_), *Gossypium raimondii* Ulbr. acc. Jianbanmian 4A (D_5_), along with 21 other diploid wild accessions (*Gossypium thurberi* Tod. D_1-5_, *Gossypium armourianum* D_2-1_, *Gossypium harknessii* D_2-2_, *Gossypium davidsonii* D_3d-27_, *Gossypium klotzschianum* Andress. D_3-k_, *Gossypium aridum* D_4_, *Gossypium gossypioides* D_6_, *Gossypium lobatum* D_7_, *Gossypium trilobum* D_8_, *Gossypium laxum* D_9_, *Gossypium turneri* D_10_, *Gossypium schwendimanii* D_11_, *Gossypium anomalum* Wawr. & Peyr. B_1_, *Gossypium stocksii* E_1_, *Gossypium longicalyx* F_1_, *Gossypium bickii* G_1_, *Gossypium. australe* G_2_, *Gossypium rotundifolium* K_2_), wild tetraploids (*Gossypium tomentosum* AD_3_, *Gossypium mustelinum* AD_4_, *Gossypium darwinii* Watt AD_5_), and the closely related distant reference sequence *Gossypioides kirkii* were incorporated (Wang et al., 2012). Detailed information regarding genome assembly versions, accession numbers, and data sources is listed in **Table 1**.

**Table 1:** *pGhFAD2-1* homologous sequences in cotton species.

Taxa	Genome	Locality/cultivar	Year first described	Length(bp)	Sequence identity to reference pGhFAD2-1 (%)	Insertion region integrity	Accession no.
Cultivated species							
*Gossypium herbaceum L*	A_1_	Africa-Asia	1753	202	12.52	Partial	CP106688.1
*Gossypium arboreum L.*	A_2_	Asia	1753	202	12.45	Partial	CM002691.1
*Gossypium hirsutum Linn.*	AD_1_	Southern Mexico	1763	1477	100	Complete	AY321158.1
*Gossypium barbadense Linn.*	AD_2_	NW South America	1753	1477	96.15	Complete	CM018227.1
*Gossypium tomentosum*	AD_3_	Hawaiian Islands	1865	1477	95.89	Complete	CM017635.1
*Gossypium mustelinum*	AD_4_	NE Brazil	1907	1478	95.89	Complete	CM017661.1
*Gossypium darwinii Watt*	AD_5_	Galapagos Islands	1907	1477	96.09	Complete	CM017713.1
Wild species							
*Gossypium anomalum Wawr. & Peyr.*	B_1_	Africa	1860	202	12.58	Partial	CM033496.1
*Gossypium thurberi Tod.*	D_1-5_	Mexico and SW U.S.	1854	1544	87.29	Complete	CM013390.1
*Gossypium armourianum*	D_2-1_	Mexico	1933	1471	88.20	Complete	CM025244.1
*Gossypium harknessii*	D_2-2_	Mexico	1889	1435	86.90	Complete	CM025231.1
*Gossypium_davidsonii*	D_3d-27_	Mexico	1873	1476	89.50	Complete	CM025218.1
*Gossypium klotzschianum Andress.*	D_3-k_	Galapagos Islands	1853	1476	89.50	Complete	CM025205.1
*Gossypium aridum*	D_4_	Mexico	1911	1468	87.55	Complete	CM025192.1
*Gossypium raimondii Ulbr.*	D_5_	Peru	1932	1480	95.37	Complete	CM001752.1
*Gossypium gossypioides*	D_6_	Mexico	1913	1449	87.22	Complete	CM025153.1
*Gossypium lobatum*	D_7_	Mexico	1956	1520	88.57	Complete	CM025283.1
*Gossypium trilobum*	D_8_	Mexico	1824	1478	92.05	Complete	CM025166.1
*Gossypium laxum*	D_9_	Mexico	1972	1478	85.31	Complete	CM025270.1
*Gossypium turneri*	D_10_	Mexico Northwest	1978	1476	88.98	Complete	CP032583.1
*Gossypium schwendimanii*	D_11_	Mexico Michoacan	1987	1476	89.31	Complete	CM025257.1
*Gossypium stocksii*	E_1_	Arabia	1874	88	5.8	Partial	CM035632.1
*Gossypium longicalyx*	F_1_	Arabia	1958	228	12.45	Partial	CM047007.1
*Gossypium bickii*	G_1_	Australia	1910	50	3.13	Partial	CP106709.1
*Gossypium australe*	G_2_	Australia	1858	50	3.06	Partial	SMMG02000001.1
*Gossypium rotundifolium*	K_2_	Western Australia	1992	235	10.23	Partial	CP106734.1
*Gossypioides kirkii*	other	Southeast Africa	1947	425	13.62	Partial	CP032255.1

### 2.2 Identification and extraction of *pGhFAD2–1* homologous sequences

To comprehensively trace the insertion and retention status of the *pGhFAD2–1* locus across the *Gossypium* phylogeny, local BLASTN searches were performed using TBtools v1.120 (Chen et al., 2020). The full-length exonic genomic DNA sequence of *pGhFAD2–1* from the *Gossypium hirsutum* TM-1 genome served as the query against the local genomic databases of the other 26 species and the distant reference sequence. The screening criteria for homologous sequences were rigorously set with the following thresholds: E-value ≤1e-10, sequence identity ≥70%, alignment coverage ≥50%, word size = 11, match score = 2, and mismatch penalty = -3. To unambiguously distinguish true orthologous sequences from other paralogous copies within the expanded *FAD2* gene family, the highly conserved flanking sequences (2,000 bp upstream and downstream of the *pGhFAD2–1* locus in TM-1) were utilized as syntenic anchors to accurately demarcate the conserved genomic blocks. Only sequences located within these conserved syntenic blocks were retained as bona fide orthologs, with paralogous copies completely excluded (Charif and Lobry, 2007). Subsequently, the strictly orthologous sequences, including the regions exhibiting presence/absence variations (PAVs) of the large insertion fragment, were precisely extracted based on their genomic coordinates for downstream analyses.

### 2.3 Sequence alignment and structural motif analysis of *pGhFAD2–1* homologous sequences

Multiple sequence alignments of the *pGhFAD2–1* homologous sequences were initially generated using DNAMAN v9.0 software (Wang, 2016) and subjected to rigorous manual refinement to eliminate ambiguous regions and uninformative alignment gaps. The resulting alignments were subsequently formatted and visualized utilizing the ESPript 3.0 server (Gouet et al., 2003). Conserved motifs across the 27 homologous sequences were predicted using the MEME Suite v5.5.4 (Bailey et al., 2009). For *cis*-acting regulatory element analysis, the exonic genomic DNA sequences of *pGhFAD2–1* orthologs were submitted to the New PLACE database (Higo et al., 1999) for element prediction. The integrated visualization of these structural features was performed using TBtools v1.120 (Chen et al., 2020).

### 2.4 Molecular evolutionary analysis

To comprehensively evaluate the evolutionary constraints and substitution patterns of the *pGhFAD2–1* locus across divergent *Gossypium* species, a series of molecular evolutionary analyses were conducted based on the refined multiple sequence alignments manually curated in the previous section. The closely related species *Gossypioides kirkii* was utilized as the distant reference sequence only for the verification of the lineage-specific distribution of this locus, and was not used for phylogenetic rooting or divergence time estimation., as it does not meet the core criterion for a valid outgroup (possessing intact orthologous sequences with the inner group) defined by classic phylogenetic systematics studies (Hillis et al., 1996; Nixon and Carpenter, 1993).Prior to phylogenetic reconstruction, the optimal nucleotide substitution model (GTR+G+I) was automatically recommended by the ModelFinder algorithm implemented in the IQ-TREE software (Kalyaanamoorthy et al., 2017; Nguyen et al., 2015). This algorithm identifies the best-fit substitution model by calculating and comparing the Bayesian Information Criterion (BIC) scores among various candidate models according to the principle of statistical optimality. The GTR+G+I model is a highly versatile and classic model for nucleotide phylogenetic analysis. It explicitly accounts for unequal base frequencies, differential base substitution rates, among-site rate heterogeneity (G, Gamma distribution), and the proportion of invariable sites (I), which perfectly matches the data characteristics of our sequence-based evolutionary inferences. Divergence times were estimated using the RelTime-ML method implemented in MEGA 11 v11.0.13 (Tamura et al., 2021) under a relaxed molecular clock model. Established divergence nodes (~10 MYA for *Gossypium*-*Gossypioides* divergence and ~5 MYA for A-D genome divergence) were utilized as calibration points (Pathak et al., 2023; Wang et al., 2022). The subsequent allopolyploidization event denoted as WGD in the motif analysis was phylogenetically demarcated at the specific divergence nodes distinguishing the allotetraploid At and Dt subgenomes from their respective diploid ancestors. Furthermore, to characterize the structural features of the exogenous insertion, transposable elements (TEs) and repetitive sequences within the 1,221-bp fragment were identified using RepeatMasker (Smit et al., 2013-2015) against the Repbase library.

To assess the equality of evolutionary rates among different subgenomic lineages (D/AD genome lineages harboring the intact *pGhFAD2–1* locus vs. non-D genome lineages with only fragmented homologous sequences), Tajima’s relative rate test was performed (Tajima, 1993), with *p* < 0.05 set as the threshold for significant rate heterogeneity. The interspecific evolutionary divergence (base differences per site) was estimated using the p-distance method with 1,000 bootstrap replicates (Liu et al., 2001). In addition, the homogeneity of substitution patterns between the two lineage groups was strictly evaluated using a Monte Carlo test (1,000 replicates) to estimate *P*-values, with *p* < 0.05 set as the significance threshold (Kumar and Gadagkar, 2001).

Finally, to infer the evolutionary dynamics and stability of the *pGhFAD2–1* locus within the D-genome lineage, the mismatch distribution (pairwise sequence differences) was calculated using DnaSP v5.10.01 (Librado and Rozas, 2009). Following the established evolutionary framework for plant lineage-specific loci, a multimodal distribution of mismatch frequencies indicates that the locus has remained evolutionarily stable under consistent selective constraints over time, whereas a unimodal distribution signifies recent rapid sequence diversification. The outputs from these evolutionary analyses were subsequently visualized as heatmaps utilizing TBtools v1.120 (Chen et al., 2020) and annotated using the *ggtree* package in R (Yu et al., 2017).

### 2.5 Histological staining and transmission electron microscopy

Histological localization of lipid droplets was conducted following our previously established protocols (Chen et al., 2025; Liu et al., 2019). Briefly, fresh cottonseed specimens were fixed in FAA solution at 4 °C for 2 h, followed by standard dehydration and paraffin embedding. Sections (5 μm thickness) were deparaffinized and co-stained with the neutral lipid-specific fluorescent dye Nile Red (0.16 μg/mL) and toluidine blue (0.5% w/v) for 20 min in the dark. Imaging and subsequent morphometric quantification were performed using a confocal microscope equipped with CaseViewer software. For TEM observation, fresh tissue blocks (~ 1 mm³) were fixed in 1% osmium tetroxide for 7 h, dehydrated through a graded ethanol series, and embedded in an 812-acetone resin mixture. Ultrathin sections (60–80 nm) were double-stained with uranyl acetate and lead citrate prior to ultrastructural observation and image acquisition under a transmission electron microscope.

### 2.6 Cottonseeds oil and fatty acid composition analysis

Cottonseed sampling at different developmental stages was performed as previously described (Ma et al., 2021). Seed oil was extracted and quantified according to Liu et al. (2019) and Ma et al. (2021). For fatty acid composition analysis, fatty acid methylation was performed using the KOH-methanol method, and qualitative and quantitative analyses were conducted using a GCMS-QP2020 system. The relevant detailed procedures were implemented according to Liu et al. (2019) and Ma et al. (2021).

### 2.7 Statistical analysis

Data processing and statistical analyses were performed using Microsoft Excel 2019 and SPSS 18.0 (SPSS, Chicago, USA). Phenotypic differences including total oil content and fatty acid components were analyzed using independent samples t-tests for pairwise comparisons, and one-way ANOVA followed by Tukey’s *post-hoc* test for multiple comparisons. The means of different species were compared at the 0.05 probability level, with exact *P*-values provided for specific pairwise phenotypic comparisons (Harms and Thornton, 2013).

## 3 Results

### 3.1 Comprehensive phylogenetic analysis and divergence time estimation of *pGhFAD2-1*

As previously established, the functional lncRNA *pGhFAD2–1* is specifically retained in D-genome lineages. To trace the precise evolutionary origin of this lineage-specific locus, we conducted a comprehensive phylogenomic analysis. Using the closely related species *Gossypioides kirkii*—a widely recognized distant reference sequence for *Gossypium* phylogenetic studies—we reconstructed a Maximum Likelihood (ML) phylogenetic tree of *pGhFAD2–1* homologous sequences under the optimal GTR+G+I nucleotide substitution model, with 1,000 bootstrap replicates. The reliability of the tree topology was supported by high bootstrap values (>80%) at all major nodes, indicating a strong phylogenetic signal during the evolutionary inference. This phylogenetic tree was constructed only to display the sequence homology and lineage-specific distribution characteristics of *pGhFAD2–1* homologous sequences across *Gossypium* species, not for resolving the deep phylogenetic relationships of different cotton genome groups.

**Figure 1:**
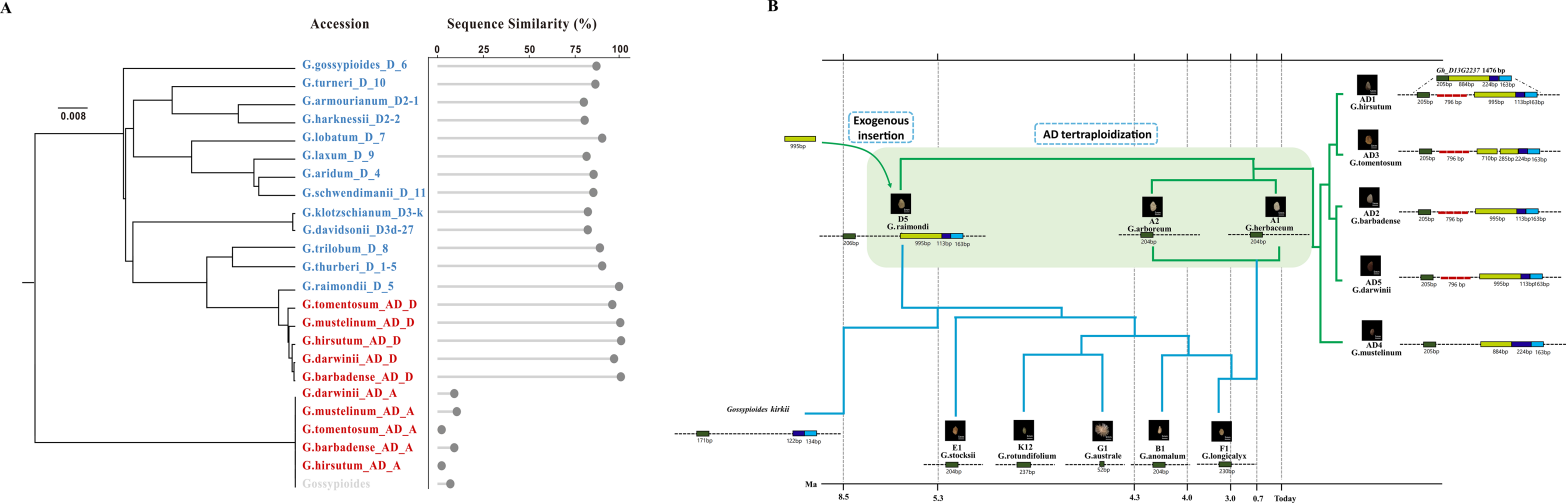
Evolution of *pGhFAD2–1* in different cotton species. **(A)** Maximum Likelihood (ML) phylogenetic tree of *pGhFAD2–1* homologous sequences from D-genome cotton species, constructed using the GTR+G+I nucleotide substitution model with 1000 bootstrap replicates, using *Gossypioides kirkii* as the distant reference sequence. Bootstrap values >50% are shown at the nodes. **(B)** Time-calibrated divergence tree of cotton species, estimated using the RelTime-ML method in MEGA 11 with established *Gossypium* divergence time nodes (10 MYA for *Gossypium*-*Gossypioides* divergence, 5 MYA for **(A–D)** genome divergence) for calibration, referring to the validated evolutionary framework reported by Pathak et al. (2023) and Wang et al. (2022).

Sequence comparisons revealed that the sequence identity of the *pGhFAD2–1* locus in the examined D-genome species (encompassing D-genome diploids and the Dt subgenome of allotetraploids) consistently exceeded 85%, utilizing the *Gossypium hirsutum* TM-1 sequence as the reference. This high sequence conservation aligned with the tree topology, where all intact D-lineage homologous sequences clustered together within a core evolutionary clade. In contrast, the At subgenome of AD-genome cotton species retained only highly fragmented remnant sequences. Due to this severe sequence degradation, the At-subgenome remnants exhibited considerable genetic distance from the D-lineage clade and clustered into a distinct, distant branch (**Figure 1A**). This topology corroborates that the intact *pGhFAD2–1* locus is exclusively a lineage-specific evolutionary innovation of the D-genome.

To delineate the evolutionary timeframe of this locus, we integrated a relaxed molecular clock model for divergence time estimation. Previous comparative genomics studies have established that the major evolutionary divergence between the *Gossypium* lineages containing the D-genome ancestor and other major lineages was initiated approximately 5 million years ago (~5 MYA) (Wang et al., 2022; Pathak et al., 2023). Our time-calibrated phylogeny precisely coincided with this major historical node. These phylogenomic data suggest that the *pGhFAD2–1* locus is not an ancient retention from the common ancestor of *Gossypium*, but originated *de novo* during the early divergence stage of these two major *Gossypium* lineages approximately 5 MYA (**Figure 1B**). This estimation provides a definitive evolutionary timeframe for the origination of this functional lncRNA.

### 3.2 Structural evolution reconstructed by tandem duplication and exogenous insertion

**Figure 2:**
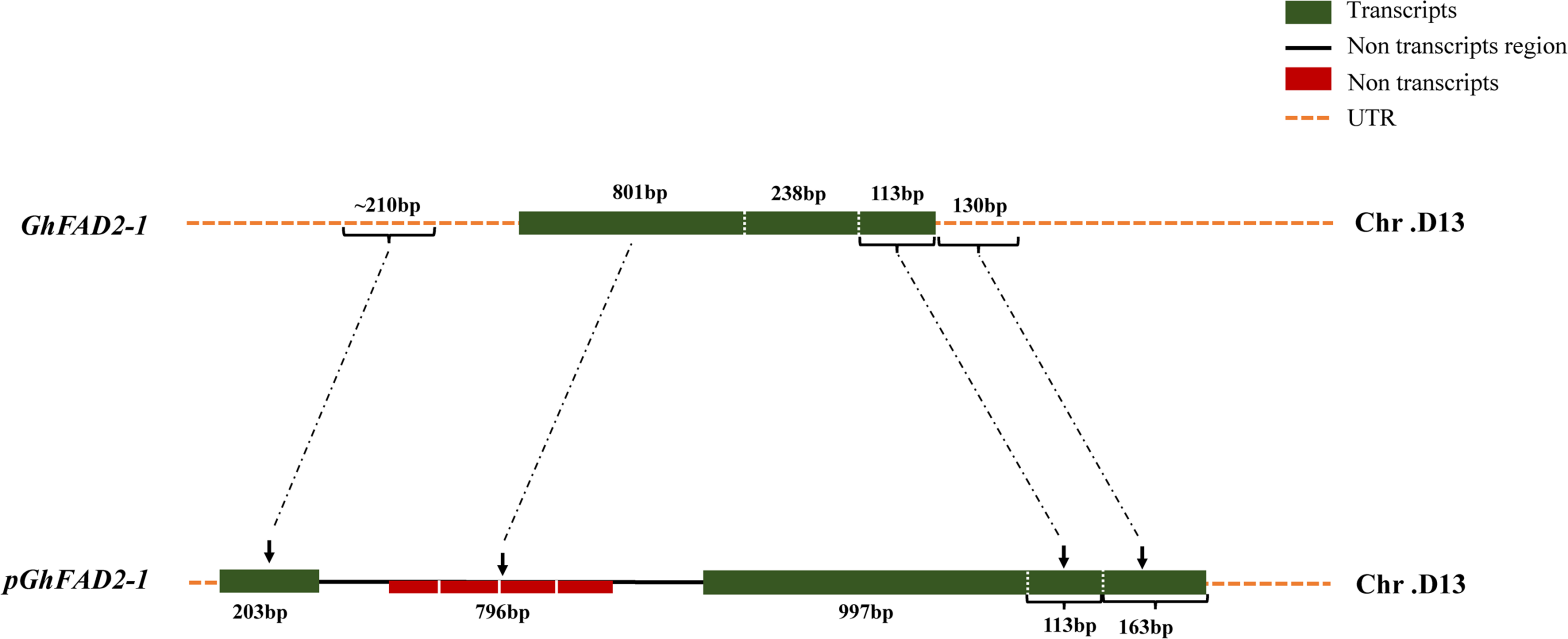
Structural alignment of *pGhFAD2–1* and its parental gene *GhFAD2-1D*. Schematic diagram showing the sequence structure comparison and homologous correspondence (dashed arrows) between the parental protein-coding gene *GhFAD2-1D* (upper panel) and its derived lncRNA *pGhFAD2-1* (lower panel) on *Gossypium* chromosome D13. Sequence annotations: green blocks = mature transcript regions; black solid lines = intergenic non-transcribed regions; red blocks = duplicated sequences from the transcribed region of *GhFAD2-1D* that lost transcriptional activity in *pGhFAD2-1*; orange dashed lines = UTRs. The length of each core structural domain is labeled above the corresponding region.

Using the full-length exonic genomic DNA sequence of *pGhFAD2–1* as a query, its homologous sequences were comprehensively identified across 27 cotton species. Genomic localizations revealed that *pGhFAD2–1* is tandemly adjacent to the functional *GhFAD2-1D* gene on the chromosome, representing a direct product of an *in situ* tandem duplication of its parental protein-coding gene *GhFAD2-1D*. Fine-scale structural alignments further demonstrated that the full-length *pGhFAD2–1* mature RNA transcript (1,476 nt) is transcribed and reorganized from four distinct genomic DNA blocks (203 bp, 997 bp, 113 bp, and 163 bp in length, respectively), whereas the adjacent GhFAD2-1D locus primarily comprises three coding exons (801 bp, 238 bp, and 113 bp) (**Figure 2**; **Supplementary Figure 1**). As summarized in **Table 1**, the full-length intact *pGhFAD2–1* locus was exclusively detected in D-genome diploid and AD-genome allotetraploid species, whereas homologous sequences in other wild species (A, B, E, F, G, and K genomes) exhibited structural fragmentations or complete deletions.

Based on these structural divergences, we further elucidated the mechanistic origin of this lncRNA. Concomitant with the *in situ* tandem duplication, the *pGhFAD2–1* locus underwent structural rearrangements, most notably a large exogenous sequence insertion. Specifically, a 1,221-bp exogenous DNA sequence was inserted into the duplicated coding region. To elucidate the exact origin of this inserted fragment, we performed a comprehensive genome-wide sequence homology search and structural analysis. Strikingly, our results revealed that this 1,221-bp exogenous sequence shares 86.69% sequence identity with a specific uncharacterized intergenic region located on chromosome D11 within the D-subgenome of *Gossypium hirsutum* (AD1) (physical coordinates: ChrD11:43912970-43913997; **Supplementary Figure 2**). The accumulated sequence divergence (~13%) between the inserted fragment and its donor locus is consistent with independent sequence evolution following the ancient insertion event (~5 MYA). Furthermore, sequence characterization confirmed that this fragment lacks typical transposable element (TE) features. This crucial evidence strongly indicates that the insertion did not emerge via canonical transposon mobilization, but rather originated from an inter-chromosomal ectopic recombination or genomic sequence capture event originating from the D11 chromosome, which has been stably conserved throughout the allotetraploidization process. During RNA splicing and maturation, a specific splicing event occurred within this inserted sequence: a 224-nt intronic sequence was excised, while the remaining 997-nt segment was retained as the core scaffold of the mature lncRNA. This insertion directly disrupted the original 238-bp core coding segment inherited from the ancestral *GhFAD2-1D* on the genome, leading to the pseudogenization of the duplicated coding sequence, as it lost the third conserved histidine box essential for *FAD2* enzymatic activity. Further sequence comparisons revealed that the 203-bp genomic segment in *pGhFAD2–1* corresponds to a highly conserved region located upstream of the *GhFAD2* locus across all *Gossypium* species. Intriguingly, while the 801-bp 5’ coding region of *GhFAD2-1D* is physically present at the *pGhFAD2–1* genomic locus, it has lost transcriptional activity and degenerated into a silent region. In contrast, the 113-bp terminal segment remains highly conserved and maintains robust transcriptional activity within *pGhFAD2-1*. In addition, a 130-bp non-transcribed downstream sequence of *GhFAD2-1D* evolved into a 163-nt transcriptional component in *pGhFAD2-1*. Collectively, these structural variations redefined precise transcriptional boundaries (initiating exclusively at an ‘A’ site and terminating at a ‘G’ site), ultimately driving the transformation of a duplicated coding gene into a novel non-coding transcript.

### 3.3 Alteration of *cis*-regulatory elements driven by structural variation

**Figure 3:**
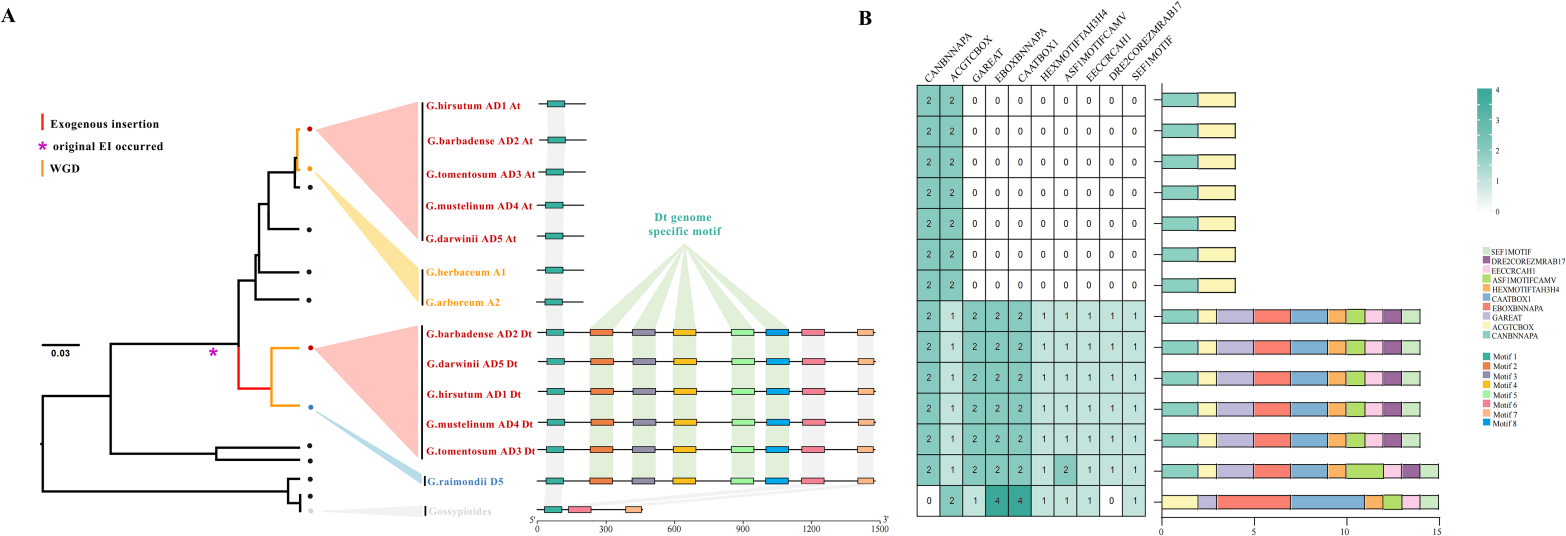
Analysis of conserved motif and Cis-acting elements of *pGhFAD2–1* in different cotton species. **(A)** The phylogenetic relationships of different cotton species based on *pGhFAD2–1* sequence. Red lines highlight the species that carry the full-length entire *pGhFAD2–1* sequence; yellow lines indicate the clustering of the At-subgenome of allotetraploid cotton species. The purple asterisk marks the critical node of genetic differentiation of *pGhFAD2-1*, with key evolutionary events annotated: EI, Exogenous Insertion; WGD, Whole-Genome Duplication. Based on sequence analysis, the sequences from different cotton species were divided into 8 conserved motifs, which are represented by different colors in the right panel. **(B)** Cis-acting regulatory element analysis of *pGhFAD2–1* and its homologous sequences. The left panel is a heatmap showing the quantitative count of core cis-acting elements, with color intensity representing the copy number of each element. The right panel shows the genomic distribution of cis-acting elements along the sequences, with different types of elements represented by unique colored blocks.

To clarify the reshaping effect of the large exogenous insertion on the regulatory potential of *pGhFAD2-1*, the 1,476-bp exonic genomic DNA sequences corresponding to the mature transcript from diverse cotton species were analyzed for cis-acting elements and conserved motifs. Among the identified 8 conserved motifs and 10 core cis-acting elements, several critical regulatory modules were specifically enriched within the D-lineage-specific inserted sequence. Notably, the 997-bp transcribed genomic segment exclusively introduced the SEF1 (soybean embryo factor 1) binding motif, the ACGTCBOX (bZIP transcription activator RITA-1 binding motif), and the HEXMOTIF (a well-characterized cis-element associated with histone gene expression and chromatin state modification). Consequently, in wild cotton species lacking the D-genome (B, E, F, G, and K genomes), the absence of this large insertion naturally abolishes these key conserved motifs and cis-regulatory elements (**Figure 3**). Furthermore, as annotated in this phylogenetic framework, whole-genome duplication (WGD) event exclusively represents the recent allopolyploidization of the AD genome lineages. The stable retention of the inserted sequence across the Dt subgenomes confirms that the regulatory architecture of the pGhFAD2–1 locus, acquired prior to this WGD event, was highly conserved throughout the allotetraploidization process.

Remarkably, the *de novo* acquisition of this histone-related DNA motif precisely aligns with the epigenetic regulatory nature of the *pGhFAD2–1* locus. As demonstrated in our previous functional characterization (Chen et al., 2025), the mature *pGhFAD2–1* RNA transcript—specifically harboring this transcribed lineage-specific insertion—serves as a molecular scaffold to directly recruit the histone deacetylase GhHDT1. Therefore, this structural innovation exerted dual regulatory effects: it not only introduced novel DNA *cis*-regulatory elements to modulate its own transcription at the genomic level, but also provided an essential RNA-protein interaction interface for epigenetic silencing at the transcript level. This coordinated regulatory divergence illustrates how lineage-specific structural variation can rewire epigenetic layers to drive adaptive phenotypic diversification in allopolyploid crops.

### 3.4 Evolutionary dynamics and purifying selection of *pGhFAD2-1*

**Figure 4:**
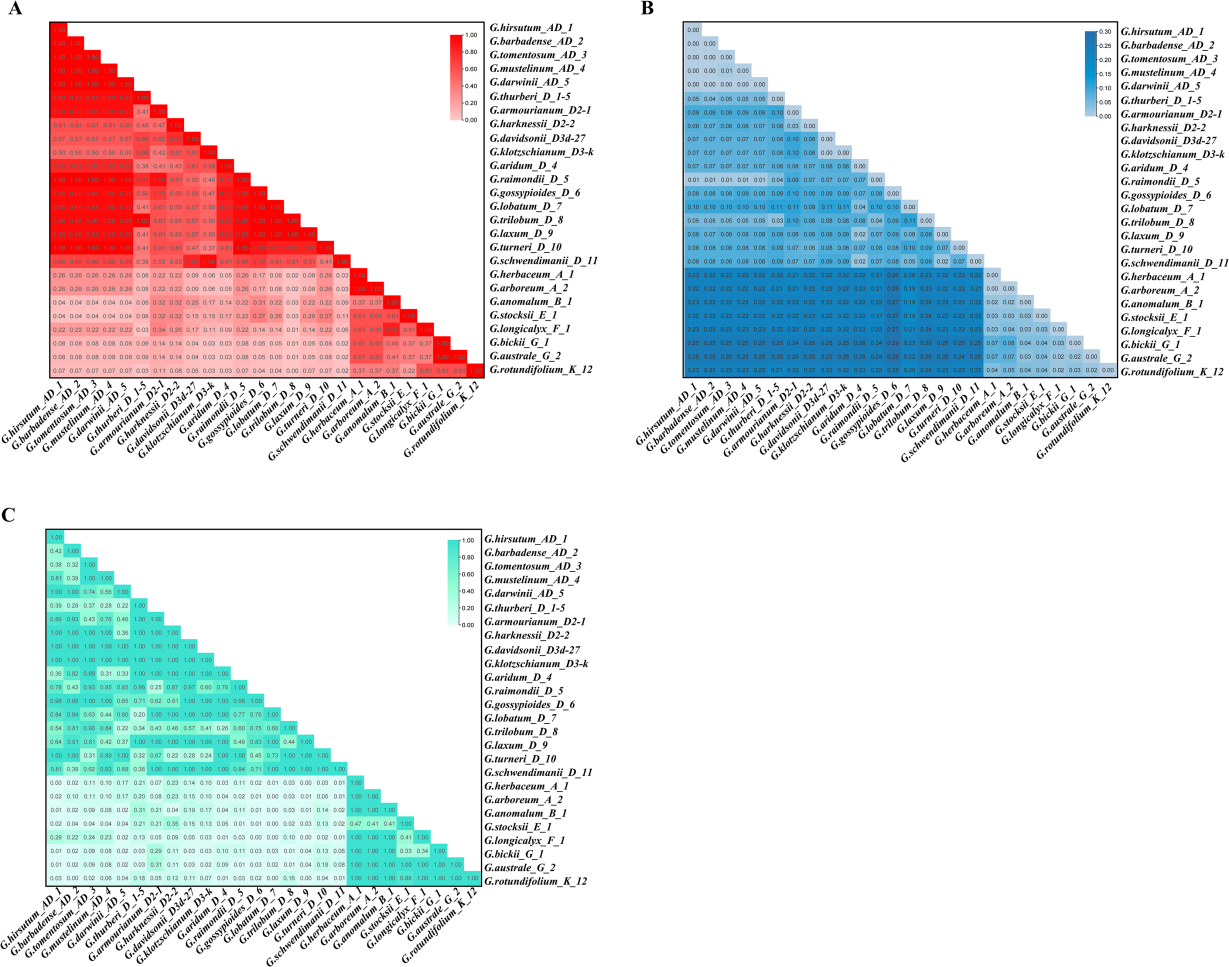
Genetic evolution analysis of *pGhFAD2–1* in different cotton species. **(A)** The relative nucleotide substitution rate in *pGhFAD2–1* from different species. *p<0.05*, inconsistent of relative evolution rates. **(B)** Estimation of evolution divergence of *pGhFAD2-1*. The numbers of base differences per site among sequences were shown; **(C)** Homogeneity test of *pGhFAD2–1* substitution pattern. *p<0.05*, significant difference in base composition biases among sequence.

To evaluate the evolutionary constraints and selective pressures acting on *pGhFAD2–1* during the lineage divergence of *Gossypium*, we analyzed the evolutionary rates and substitution patterns of this sequence across different cotton species. The results indicated that the relative evolutionary rates of *pGhFAD2–1* sequences in the AD and D genomes were significantly different from those in the A, B, E, F, G, and K genomes (*p<0.05*) (**Figure 4A**). Compared to the AD and D-genome lineages, other wild cotton species lacking the D-genome exhibited a significantly higher accumulation of base substitutions at each site, indicating substantial interspecific sequence divergence between lineages with and without the intact *pGhFAD2–1* locus (**Figure 4B**). In addition, significant differences were observed in the homogeneity of substitution patterns between these two lineage groups (**Figure 4C**). This significant difference in evolutionary rates further confirms that the intact *pGhFAD2–1* locus in D/AD genomes is subject to strong selective constraints, while the fragmented sequences in non-D genomes evolve under neutral selection without functional constraints.”

**Figure 5:**
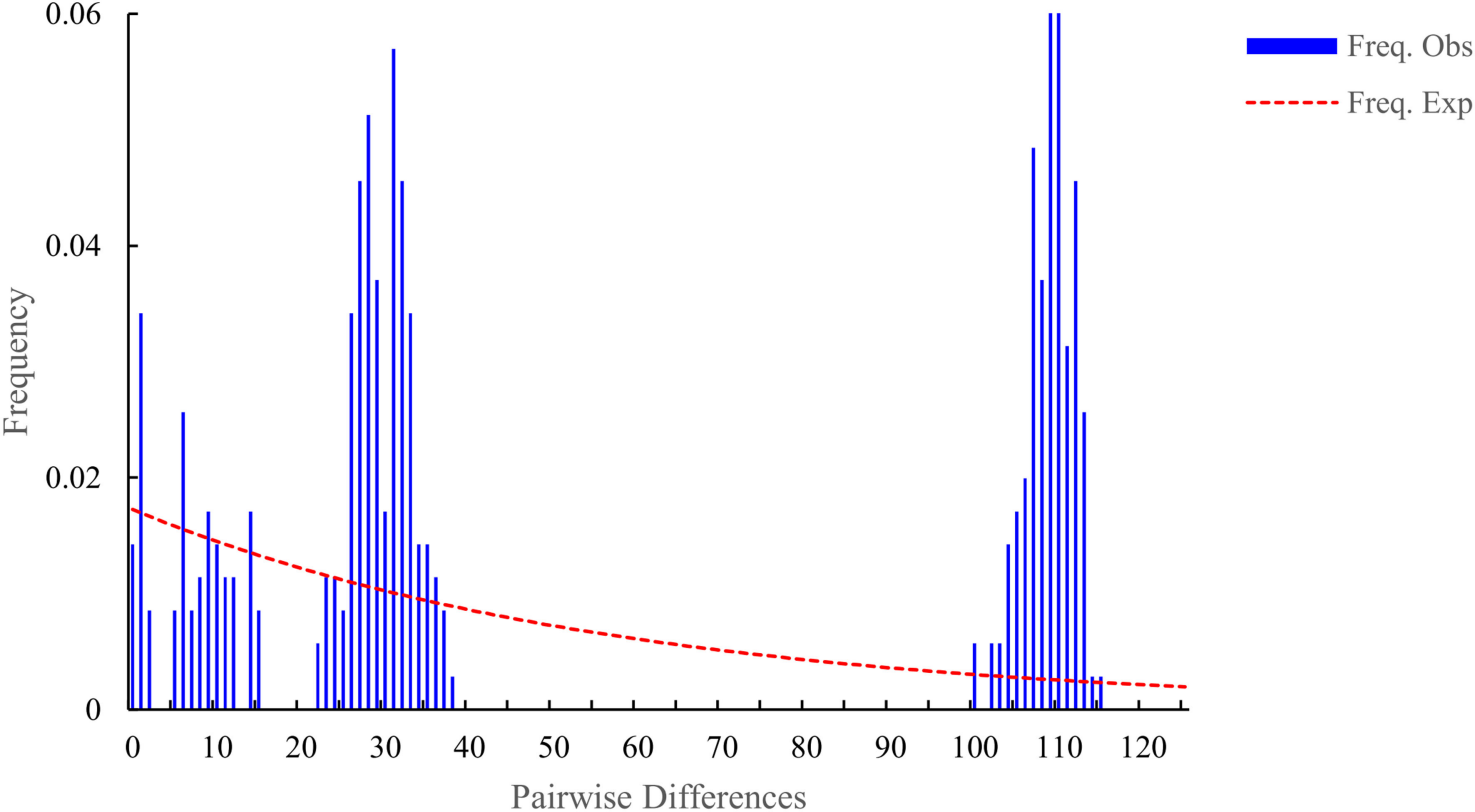
The pairwise mismatch distribution of homologous *pGhFAD2–1* in D-genome species. The multimodal pattern of the mismatch distribution indicates a relatively constant population size of D-genome species without recent severe demographic expansion.

To comprehensively evaluate the evolutionary stability of *pGhFAD2–1* following its origination in D-genome lineages, we performed a mismatch distribution analysis. The mismatch frequencies of *pGhFAD2–1* homologous sequences among D-genome species exhibited a typical multimodal distribution pattern (**Figure 5**). Consistent with established evolutionary paradigms for plant lineage-specific functional loci, this stable multimodal distribution pattern, combined with the >85% sequence identity across D-genome lineages, demonstrates that the *pGhFAD2–1* locus has been subjected to strong purifying selection to maintain its core epigenetic regulatory function, and remained highly conserved within D-genome lineages after its *de novo* origination.

### 3.5 Phenotypic divergence of lipid metabolism across representative *Gossypium* species

To investigate whether the molecular evolutionary trajectory of the *pGhFAD2–1* locus translates into phenotypic divergence, we selected five typical species representing the major evolutionary clades of *Gossypium* (diploid ancestors and allotetraploids) as research materials. These included *Gossypium herbaceum* L. (A_1_), *Gossypium arboreum* L. (A_2_), *Gossypium raimondii* Ulbr. (D_5_), *Gossypium hirsutum* L. (AD_1_), and *Gossypium barbadense* L. (AD_2_). The lipid distribution and oil content of their mature cottonseeds were phenotypically evaluated.

**Figure 6:**
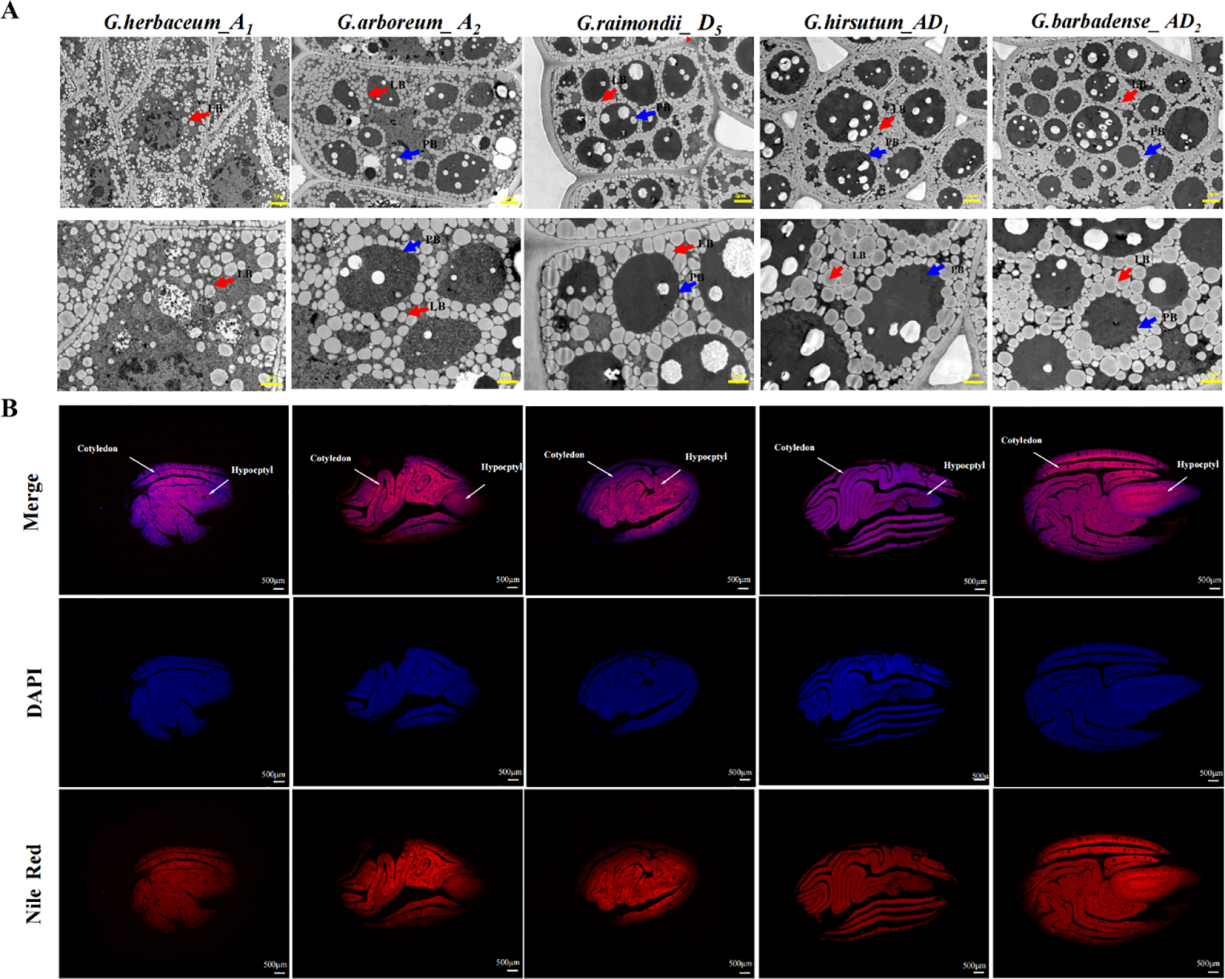
Cytological and phenotypic divergence of cottonseed lipid metabolism across representative *Gossypium* species. **(A)** Transmission electron microscopy (TEM) observation of lipid bodies (LBs, red arrows) and protein bodies (PBs, blue arrows) in mature cottonseed cotyledon cells. Scale bar=2 μm. **(B)** Nile Red fluorescence staining of LBs in mature cottonseeds. Scale bar=100 μm. Three biological replicates were performed per species for all cytological experiments.

Transmission electron microscopy (TEM) observations revealed that the distribution of lipid bodies (LBs) and protein bodies (PBs) within the A_1_ genome cottonseeds was relatively loose, whereas those in the A_2_, AD_1_, AD_2_, and D_5_ genome cottonseeds were distributed more compactly and densely (**Figure 6A**). Further Nile Red fluorescence staining indicated that, despite obvious variations in overall organ volume among different genome cottonseeds, the fluorescence intensity and spatial distribution density of LBs were fully consistent with the subsequent quantitative oil content measurements (**Figure 6B**).

Subsequently, we quantitatively determined the absolute oil content and core fatty acid compositions of the examined wild and cultivated cotton species (**Table 2**). The results demonstrated that the relative oil content of the D-genome wild cotton (D_5_) was 19.85%, which was essentially equivalent to that of the AD_1_ genome species. However, compared to the A_1_ and A_2_ genome species lacking the intact *pGhFAD2–1* locus, the oil content of D_5_ was lower by 3.4 percentage points (*p* = 0.087) and significantly lower by 9.3 percentage points (*p* = 0.003), respectively. Regarding fatty acid composition, the relative palmitic acid (C16:0) proportion in the A_1_ and A_2_ genomes was significantly lower than that in the D_5_, AD_1_, and AD_2_ genomes. Specifically, the C16:0 proportion of A_1_ was 14.23% (*p* = 0.004), 12.63% (*p* < 0.001), and 15.64% (*p* < 0.001) lower than that of D_5_, AD_1_, and AD_2_, respectively. Conversely, the oleic acid (C18:1) proportion in the A_1_ genome species was significantly higher than that in the D_5_ and AD_1_ genomes, showing an increase of 14.92% (*p* = 0.021) and 10.45% (*p* = 0.031), respectively.

**Table 2:** Total oil content and main fatty acid composition of mature cottonseeds from representative *Gossypium* species.

Taxa	Genome	Relative content (%, mean ± SD)					
		Oil content	Palmitic acid	Stearate acid	Oleate acid	Linoleate acid	Linolenic acid
			C16:0	C18:0	C18:1	C18:2	C18:3
*Gossypium hirsutum L.*	(AD)_1_	19.81 ± 0.35c	24.14 ± 0.36b	3.35 ± 0.23b	19.03 ± 0.71c	50.94 ± 0.4a	0.24 ± 0.06a
*Gossypium barbadense L.*	(AD)_2_	21.66 ± 0.63a	25 ± 0.31a	3.33 ± 0.31b	21.65 ± 0.82b	47.06 ± 0.71b	0.12 ± 0.03b
*Gossypium herbaceum L*	A_1_	20.55 ± 0.31b	21.09 ± 0.2e	4.27 ± 0.19a	21.02 ± 0.78b	50.43 ± 0.82a	0.1 ± 0.03b
*Gossypium arboreum L.*	A_2_	21.89 ± 0.32a	22.3 ± 0.62c	3.08 ± 0.13c	30.68 ± 0.8a	41.48 ± 0.67c	0.12 ± 0.03b
*Gossypium raimondii Ulbr.*	D_5_	19.85 ± 0.44bc	24.59 ± 0.97ab	4.11 ± 0.06a	18.29 ± 1.02c	50.56 ± 1.35a	0.08 ± 0.01b

Data are from three biological replicates (n=3). Different lowercase letters within a column denote significant differences (*p* < 0.05, one-way ANOVA followed by Tukey’s post-hoc test).

Data are presented as mean ± SD of three biological replicates.

In summary, the representative cotton species containing the D-genome (AD and D genomes) generally exhibited higher C16:0 proportions but significantly lower overall oil content and C18:1 proportions compared to the A-genome species. These results indicate that cotton species situated in divergent evolutionary clades (with and without the functional *pGhFAD2–1* locus) exhibit significant divergence in cottonseed lipid traits. This phenotypic divergence closely aligns with the genomic evolutionary trajectories revealed previously, providing phenotypic evidence that the lineage-specific retention of *pGhFAD2–1* is tightly associated with the adaptive divergence of cottonseed lipid metabolism, which is fully consistent with its previously validated repressive function in lipid accumulation.

## 4 Discussion

As a premier global economic crop, upland cotton (*Gossypium hirsutum* L) serves dually as a core raw material for the textile industry and a primary source of edible oil. In this context, *FAD2* (^Δ12-^fatty acid desaturase) acts as the rate-limiting enzyme catalyzing the conversion of oleic acid (C18:1) to polyunsaturated linoleic acid (C18:2), thus dictating the final ratio of unsaturated fatty acids. In our previous study, we identified that a tandemly duplicated sequence of *GhFAD2-1D* on chromosome D13 lost its protein-coding capacity and instead functions as a lineage-specific long non-coding RNA (lncRNA), designated as *pseudo-GhFAD2-1* (*pGhFAD2-1*). This lncRNA epigenetically regulates cottonseed lipid metabolism by recruiting the histone deacetylase GhHDT1 (Chen et al., 2025). However, the evolutionary origin and adaptive significance of this lineage-specific regulatory lncRNA have long remained unresolved. The present study systematically dissects the *de novo* origination of *pGhFAD2–1* approximately 5 MYA within the D-genome lineage. Our findings reveal that a single lineage-specific structural reorganization simultaneously endowed this locus with DNA-level transcriptional responsiveness and an RNA-level epigenetic interaction interface. Furthermore, the strict asymmetric retention of this locus in the Dt subgenome extends the paradigm of polyploid asymmetric evolution to non-coding regulatory elements, highlighting the evolutionary trade-offs that shape cottonseed lipid metabolism.

### 4.1 Structural variation and the *de novo* origination of *pGhFAD2-1*

Lineage-specific structural variations and tandem duplications are widely established as fundamental evolutionary engines driving the *de novo* origination of functional lncRNAs (Ulitsky, 2016; Zhao et al., 2022). Our phylogenomic data across 27 *Gossypium* species reveal that the genesis of *pGhFAD2–1* was initiated by an *in situ* tandem duplication of *GhFAD2-1D*, followed by a D-lineage-specific sequence capture (Grover et al., 2022). Crucially, our genomic tracing explicitly links this exogenous insertion to an inter-chromosomal ectopic recombination event originating from an uncharacterized intergenic region on chromosome D11. Indeed, the inter-chromosomal capture of genomic fragments is emerging as a potent, yet underappreciated, mechanism for regulatory innovation (Kaessmann, 2010).

This structural reorganization was not merely a disruptive event. Traditionally, duplicated genes that incur severe structural disruptions and frame-shifts are considered evolutionary “dead ends” destined for rapid pseudogenization and elimination from the genome (Lynch and Conery, 2000). However, emerging evolutionary paradigms demonstrate that such pseudogenized loci can occasionally be repurposed into regulatory non-coding RNAs (Cheetham et al., 2020). Our findings provide striking empirical evidence for this pseudogene-to-lncRNA transition in polyploid crops. Unlike the gradual accumulation of neutral point mutations that drives the slow functional divergence of paralogs under the canonical neofunctionalization model (Ohno, 1970; Conant and Wolfe, 2008), this single large-scale structural rearrangement event acted as an “evolutionary leapfrog.” It simultaneously pseudogenized the coding sequence, introduced novel *cis*-regulatory elements natively inherited from the captured D11 sequence, and repurposed the duplicate into an epigenetic regulatory non-coding node. By bypassing the traditional slow divergence trajectory of duplicated genes, this D11-derived inter-chromosomal sequence capture provides a compelling evolutionary model for how gene duplication coupled with ectopic genomic rearrangements can instantaneously orchestrate the *de novo* birth of complex regulatory lncRNAs within the dynamic genomes of allopolyploid crops.

### 4.2 Evolution of *pGhFAD2–1* into a lineage-specific epigenetic regulatory node

Following the lineage-specific structural innovation, a critical evolutionary question arises regarding how a pseudogenized locus rapidly acquired the capacity to function as a sophisticated epigenetic regulator. The functionalization of *de novo* originated lncRNAs typically necessitates a protracted evolutionary period, relying on the gradual accumulation of point mutations to generate functional transcriptional promoters and RNA interaction domains (Schlötterer, 2015; Statello et al., 2021). However, the molecular architecture of *pGhFAD2–1* illustrates an alternative and highly efficient trajectory. The interchromosomal sequence capture event essentially donated an intact regulatory module, conferring dual functionalities simultaneously and thereby bypassing the slow divergence trajectory of paralogs.

At the transcriptional level, lipid accumulation during seed development is finely coordinated by a complex transcriptional regulatory network (Ruuska et al., 2002; Zhao et al., 2018). Within this network, basic leucine zipper (bZIP) transcription factors recognize specific ACGTCBOX motifs to fine-tune metabolic flux (Armstrong et al., 1992; Mendes et al., 2013; Yang et al., 2023). The captured D11 sequence natively harbored this bZIP-responsive module, directly endowing the pseudogenized locus with DNA-level transcriptional competence during the critical window of seed maturation. Because the A-genome species naturally lack this ectopic insertion, the recruitment of transcription factors such as bZIPs to this genetic node is strictly confined to the D-lineage. This structural presence and absence variation elegantly explains the lineage-specific transcriptional activation of this regulatory module without the necessity to invoke independent *cis*-element evolution.

Furthermore, the functional significance of this sequence capture extends beyond typical transcriptional activation and extends directly to epigenetic remodeling. The co-captured sequence inherently contains the HEXMOTIF, which is a conserved *cis*-element linked to chromatin state modification. As validated by our previous functional experiments (Chen et al., 2025), the *pGhFAD2–1* RNA transcript strictly requires this specific exonic region to serve as a functional scaffold for recruiting the histone deacetylase GhHDT1. The concurrent acquisition of these functional motifs implies that the structural insertion event endowed the locus with an integrated and immediately active interaction platform (Ariel et al., 2014).

Collectively, this structural reorganization catalyzed a synergistic dual functionalization process. By simultaneously acquiring DNA-level responsiveness for spatiotemporal transcription and an RNA-level interface for epigenetic protein recruitment, the *pGhFAD2–1* locus was instantaneously equipped with the molecular capacity to remodel the local histone acetylation landscape and suppress cottonseed lipid biosynthesis. This integrated mechanism not only elucidates the genetic basis for the phenotypic divergence in fatty acid composition between D and A genome species but also highlights how single large-scale genomic rearrangements can rapidly assemble complex epigenetic nodes in allopolyploid crops.

### 4.3 Purifying selection and asymmetric subgenome retention of *pGhFAD2-1*

While the vast majority of recently emerged lncRNAs exhibit rapid sequence turnover and are continuously purged from the genome via neutral drift (Hezroni et al., 2015), our molecular evolutionary assessments confirm that the *pGhFAD2–1* locus has been subjected to strong purifying selection and has remained highly conserved within the D-genome lineage (Ponjavic et al., 2007). This robust conservation indicates that the structural variation and the subsequent acquisition of epigenetic regulatory capacity conferred a distinct adaptive advantage. This evolutionary trajectory perfectly exemplifies how newly emerged non-coding transcripts that become physically integrated into essential epigenetic complexes face stringent functional constraints, which actively prevent their sequence degradation (Ulitsky and Bartel, 2013). Consequently, these selective pressures dictated the conservative fixation of this locus in D-genome diploids and its subsequent vertical transmission to the Dt subgenome of allotetraploid cottons following allopolyploidization.

This asymmetric presence and absence pattern is a typical molecular imprint of the well-documented asymmetric subgenome evolution in allotetraploid cotton. Multiple pan-genomic studies have established that the Dt subgenome retained significantly more selection signatures associated with agronomic traits than the At subgenome during domestication (Zhang et al., 2015; Wang et al., 2017; Ma et al., 2018). However, these foundational models have predominantly concentrated on the asymmetric expression and retention of protein-coding genes. By elucidating the evolutionary trajectory of *pGhFAD2-1*, we extend this classic paradigm to lineage-specific non-coding regulatory elements. The exclusive retention of this locus serves as a striking example of how the Dt subgenome uniquely preserves epigenetic repressors to fine-tune highly conserved metabolic pathways inherited from diploid ancestors. This extension underscores the critical, yet previously underappreciated, role of the non-coding genome in driving the subgenomic coordination and lineage-specific adaptation of allopolyploid crops.

### 4.4 Evolutionary trade-offs in carbon allocation drive the adaptive retention of *pGhFAD2-1*

Building on the signature of strong purifying selection and asymmetric subgenomic retention of *pGhFAD2–1* characterized above, the stringent evolutionary constraints acting on this locus translate directly into macroscopic phenotypic divergence in cottonseed lipid metabolism. Our phenotypic evaluations demonstrate that total seed oil content exhibits no significant difference between the diploid *Gossypium raimondii* (D5) and the allotetraploid *Gossypium hirsutum* (AD1), both of which harbor the functional *pGhFAD2–1* module. Conversely, the overall oil content and oleic acid levels in the A-genome diploid species, which naturally lack this epigenetic repressor, are significantly higher. This clear phenotypic divergence pattern strictly corroborates the negative regulatory role of the *pGhFAD2-1*–*GhHDT1* module. This lineage-specific repression aligns with the Dt subgenome-biased genomic landscape of cotton domestication, where modern resequencing studies have identified profound selection signatures at loci associated with agronomic traits and metabolic adaptation in the Dt subgenome (Su et al., 2016; Ma et al., 2018). Furthermore, the exclusive retention of this repressive module in the Dt subgenome is highly consistent with the extensive cis- and trans-regulatory evolution observed during the domestication of allotetraploid cotton (Bao et al., 2019).

The stable retention of a lipid-suppressing locus within the D-genome lineage presents an apparent evolutionary paradox: energy-dense triacylglycerol reserves are widely recognized to confer adaptive advantages for seed germination and seedling establishment, so why has this repressive locus been maintained by strong purifying selection? This counterintuitive pattern is best interpreted through the classic framework of carbon allocation trade-offs during plant adaptation (Ding and Chen, 2018). The biosynthesis of storage lipids is a metabolically expensive process that competes for finite photosynthetic carbon precursors with alternative sink tissues. In oilseed models such as *Brassica napus*, suppressing seed lipid accumulation has been shown to inversely divert carbon flux toward competing sink components (Chapman et al., 2008). Within the evolutionary context of *Gossypium*, we propose that the *pGhFAD2–1* locus functions as an adaptive metabolic checkpoint. By restricting excessive carbon drainage into storage oils, this epigenetic module potentially facilitates a more favorable metabolic background for the development of alternative carbon-demanding structures, specifically the highly specialized seed coat trichomes (cotton fibers). While the direct quantitative contribution of this specific locus to fiber elongation remains to be precisely validated through future genetic experiments, our macro-evolutionary framework suggests that the Dt subgenome may have co-opted this ancestral metabolic constraint that arose ~5 million years ago in the D-genome ancestor to accommodate the immense carbon demands of fiber cell development.

The characterization of this lineage-specific epigenetic repressor provides a clear theoretical pathway for breaking the long-standing negative genetic correlation between cottonseed lipid metabolism and fiber development. By utilizing targeted genome editing or epigenetic perturbation to precisely decouple this metabolic checkpoint, future molecular breeding strategies could optimize carbon partitioning, facilitating the synergistic improvement of both cottonseed oil nutritional value and fiber yield in dual-purpose elite cotton varieties.

## 5 Conclusion

By integrating comprehensive evolutionary and phenotypic analyses across 27 representative *Gossypium* species, this study systematically resolves the *de novo* origination and adaptive trajectory of the lineage-specific lncRNA *pGhFAD2-1*. We demonstrate that this locus emerged approximately 5 million years ago (MYA) through an *in situ* tandem duplication of *GhFAD2-1D*, followed rapidly by an interchromosomal sequence capture event unique to the D-genome lineage. Rather than succumbing to pseudogenization, this structural innovation catalyzed an instantaneous dual functionalization. By simultaneously acquiring cis-regulatory elements at the DNA level and a *GhHDT1*-binding scaffold at the RNA level, the locus was elegantly transformed into a critical epigenetic repressor. Subsequently, this newly assembled regulatory module experienced strong purifying selection, dictating its conserved fixation in D-genome diploids and its asymmetric retention within the Dt subgenome of allotetraploid cottons. This exclusive subgenomic retention reflects a macroscopic evolutionary trade-off in carbon resource allocation, effectively restricting excessive lipid biosynthesis to prioritize the metabolic demands of fiber development. Ultimately, these findings establish a comprehensive evolutionary paradigm for the *de novo* origination of functional lncRNAs in polyploid crops. Furthermore, they highlight the *pGhFAD2–1* metabolic checkpoint as a premier molecular target for decoupling lipid and fiber developmental networks, providing a robust theoretical foundation for breaking the long-standing negative genetic correlation between fiber yield and seed oil content, and enabling the synergistic genetic improvement of dual-purpose elite cotton varieties.

## Data Availability

The original contributions presented in the study are included in the article/**Supplementary Material**. Further inquiries can be directed to the corresponding authors.

## Supplementary material

Supplementary Figure 1 Detailed nucleotide sequence annotation and structural features of the *pGhFAD2-1.* The full-length cloned transcript sequence (1,476 nt) is highlighted in yellow. The precise transcription start site (TSS) and transcription termination site (TTS) are indicated in bold red letters. Sequences sharing homology with the ancestral *GhFAD2–1* gene are underlined. The spliced intronic region is characterized by canonical GT-AG splice donor and acceptor sites. The 1,221-bp exogenous insertion, presumably originating from an ectopic genomic region, is denoted in red text.

Supplementary Figure 2 Sequence alignment between the 1,221-bp exogenous insertion of *pGhFAD2–1* and its homologous genomic region.
