# Correction: Evolutionary origin and asymmetric subgenomic retention of the lncRNA *pGhFAD2–1* that regulates cotton lipid metabolism

**DOI:** 10.3389/fpls.2026.1878870

**Published:** 2026-06-12

**Authors:** Haihong Chen, Xuan Liu, Ni Yang, Zhaolong Gong, Fenglei Sun, Shiwei Geng, Juyun Zheng, Shuaishuai Qian, Junduo Wang, Yajun Liang

**Affiliations:** 1 National Cotton Engineering Technology Research Center, Cotton Research Institute of Xinjiang Uyghur Autonomous Region Academy of Agricultural Sciences, Wulumuqi, Xinjiang, China; 2 Agricultural Genomics Institute at Shenzhen, Chinese Academy of Agricultural Sciences, Shenzhen, China

**Keywords:** cottonseed oil, evolutionary dynamics, *Gossypium*, pGhFAD2-1, structural variation

Author “Feng Liu” has voluntarily requested to be removed from the author list of the published article. The author has confirmed that he did not make substantial contributions to the design, execution, or writing of this study. “Feng Liu” has also been removed from the corresponding author list. Affiliation 3 “Key Laboratory of Oasis Eco-agriculture, College of Agriculture, Shihezi University, Shenzhen, China” has been removed as a result of this author list change.

The correct author list reads:

“Haihong Chen1†, Xuan Liu2†, Ni Yang1†, Zhaolong Gong1, Fenglei Sun1, Shiwei Geng1, Juyun Zheng1, Shuaishuai Qian1, Junduo Wang1* and Yajun Liang1*

1 National Cotton Engineering Technology Research Center, Cotton Research Institute of Xinjiang Uyghur Autonomous Region Academy of Agricultural Sciences, Wulumuqi, Xinjiang, China,

2 Agricultural Genomics Institute at Shenzhen, Chinese Academy of Agricultural Sciences, Shenzhen, China”

The **Author Contributions** statement has been corrected to read:

“HC: Writing – original draft, Writing – review & editing. XL: Conceptualization, Data curation, Formal analysis, Funding acquisition, Investigation, Methodology, Project administration, Resources, Software, Supervision, Validation, Visualization, Writing – original draft. NY: Conceptualization, Data curation, Formal analysis, Funding acquisition, Investigation, Methodology, Project administration, Resources, Software, Supervision, Validation, Visualization, Writing – original draft. ZG: Writing – review & editing. FS: Writing – review & editing. SG: Writing – review & editing. JZ: Writing – review & editing. SQ: Writing – review & editing. JW: Writing – review & editing. YL: Writing – review & editing.”

There was a mistake in the caption of **Figure 6** as published.

The phenotypic images contained an inadvertent labeling error. The corrected **Figure 6** appears below.

**Figure 6:**
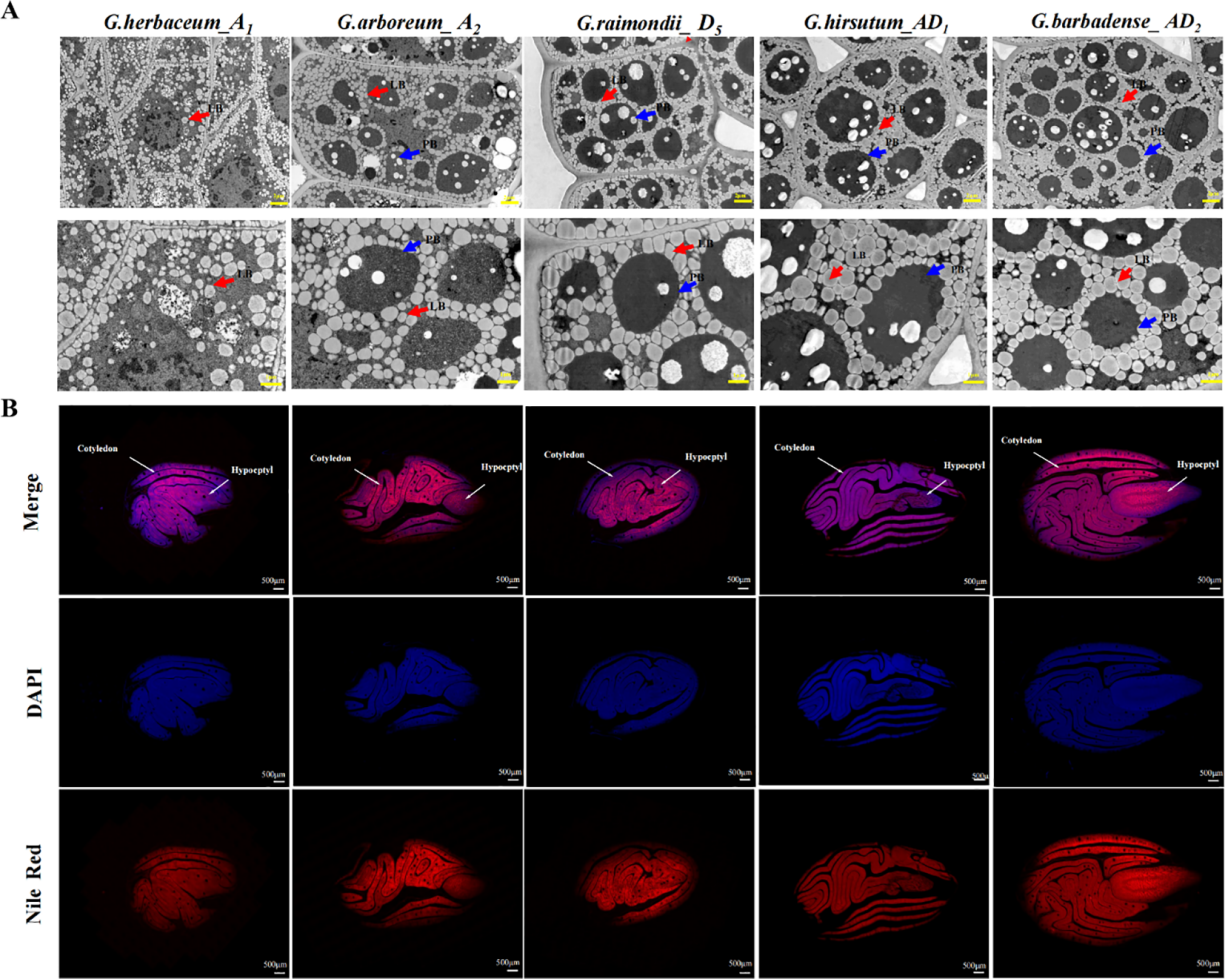


The original version of this article has been updated.

