# Supplementary figures and images for: Evolutionary origin and asymmetric subgenomic retention of the lncRNA pGhFAD2–1 that regulates cotton lipid metabolism
Source: Front Plant Sci. 2026 May 8;17:1841757. doi: 10.3389/fpls.2026.1841757 (PMC13194438; doi:10.3389/fpls.2026.1841757)

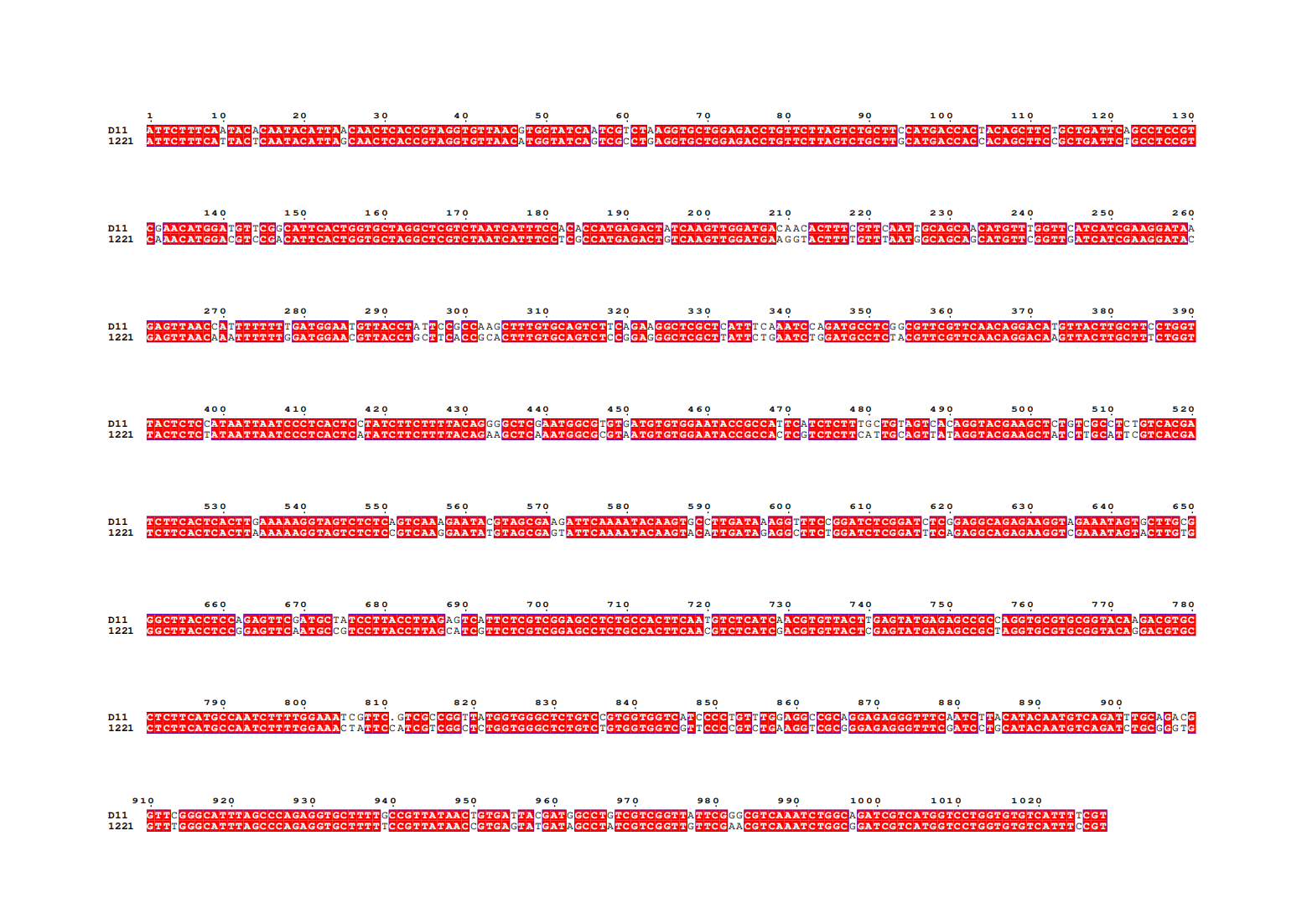

Supplement: Supplementary Figure 1 — Detailed nucleotide sequence annotation and structural features of the pGhFAD2-1. The full-length cloned transcript sequence (1,476 nt) is highlighted in yellow. The precise transcription start site (TSS) and transcription termination site (TTS) are indicated in bold red letters. Sequences sharing homology with the ancestral GhFAD2–1 gene are underlined. The spliced intronic region is characterized by canonical GT-AG splice donor and acceptor sites. The 1,221-bp exogenous insertion, presumably originating from an ectopic genomic region, is denoted in red text. [file Image1.tif]
