# Supplementary material for: Evolutionary origin and asymmetric subgenomic retention of the lncRNA pGhFAD2–1 that regulates cotton lipid metabolism
Source: Front Plant Sci. 2026 May 8;17:1841757. doi: 10.3389/fpls.2026.1841757 (PMC13194438; doi:10.3389/fpls.2026.1841757)
Supplement: Supplementary Figure 2 — Sequence alignment between the 1,221-bp exogenous insertion of pGhFAD2–1 and its homologous genomic region. [file DataSheet1.doc]

Transcription starting site of *GhFAD2-1D*

5’UTR intron of *GhFAD2-1D*

*GhFAD2-1D,1152bp, no intron, encoding 383AA*

Transcription starting site of *pGhFAD2-1*

**T**GGCGTTAAACTGCTTTCTTTAAA--5'UTR intron(1100nt)**--**GCCAGCAAA**ATG**--**CDS:1152nt**(383AA)--**TAA**--**1205nt**—**A**GCAAGAAT CATCAGAATAAAAAAAAAAGGGAAAAGA CCCCCATCCCCACTGATTCTGGTCTTGCTTCCCAACACGTAGCATCTTACCAAAAAACCTCAAATCCACCACT CCTCTATTTCAAAACCCCCCTTCGCTCCACTCCACTCGCCCAAAACCAACACGCCTTCTTTAATCACCAGGCGTTAAATTGCTTTCTTTAAAGG**T**--**1211nt**--ATGGGTGTCGGTGGTAGGATGGTAGTTCACGGTAAAAAGAATAAGGAAAACCGAGGTTGGGTCAATCGAGTTTCGATCAAGAAGCCTCCGTTTACGCTCAGTCAGATCAAGCAAGACATTCTGCCCCACTGTTTTCGTCGCTCCCTCCTTCGATCCTTCTCCTACGTGGTCCATGACCTATGCTTAGCCTCTCTCTTTTACTACATTGCAACATCATATTCACTTTCTCCCACATCCCTTTTCCTACATTGCTTGGCCTGTCTATTGGGTTCTCCAAGGTTGCATCCTCACCGGTGTTTGGGTCATCGCACACGAGTGCGGTCACCACGCTTTCAGTGACTACCAATGGGTTGACAACACCGTCGGGTTGATCCTTCACTCCACCCTTTTAGTCCCGTACTTCTCGTGGAAAATCAGTCACCGCCGTTACCACTCAAACACCGGTTCTATGGAGCGTGACAAAGTATTCGTGCCCAAACCCAAGTCTAAATTATCATGCTTTGCGAAATACTTAAACAATCCACCCGGTCGAGTTCTATCTCTTGTAGTCACATTGACTCTAGGTTGGCCTATGTACTTAGCCTTCAACGTTTCAGGTCGATACTATGATCAATTAGCTTCCCACTATAATGGCCCCATTTTCTCTGATTGCGAGAGGCTACAAGTTTACATCTCCGATGCTAGTATATTTACGGTAATTTATGTACTTTATAAGATTACTGCAACAAAAGGGCTGGCTTGGCTTTTATGCACTTATGGGGTACCTCTACTTATTGTGCATGCCTTCCTTGTGTTGCTAACTTTGTTAAGAGTTAAGAGTATAGATAAGAACTGTGCTAGCTAACTTTGTTATTGAGCTAGCCTATTAGCTGTTAGTTTGTTATTCAAGCAATTAAGCTGTTAGTAATACAAGTTACTCGTATATTCTATAAATACCATTGTTATAATGTACGAACTTGATATACAAGACTTAATACAAAGAATTCTTTCATTACTCAATACATTAGCAACTCACCGTAGGTGTTAACATGGTATCAGTCGCCTGAGGTGCTGGAGACCTGTTCTTAGTCTGCTTGCATGACCACCACAGCTTCCGCTGATTCTGCCTCCGTCAAACATGGACGTCCGACATTCACTGGTGCTAGGCTCGTCTAATCATTTCCTCGCCATGAGACTGTCAAGTTGGATGAAGGTACTTTTGTTTAATGGCAGCAGCATGTTCGGTTGATCATCGAAGGATACGAGTTAACAAATTTTTTGGATGGAACGTTACCTGCTTCACCGCACTTTGTGCAGTCTCCGGAGGGCTCGCTTATTCTGAATCTGGATGCCTCTACGTTCGTTCAACAGGACAAGTTACTTGCTTTCTGGTTACTCTCTATAATTAATCCCTCACTCATATCTTCTTTTACAGAAGCTCAAATGGCGCGTAATGTGTGGAATACCGCCACTCGTCTCTTCATTGCAGTTATAGGTACGAAGCTATCTTGCATTCGTCACGATCTTCACTCACTTAAAAAAGGTAGTCTCTCCGTCAAGGAATATGTAGCGAGTATTCAAAATACAAGTACATTGATAGAGGCTTCTGGATCTCGGATTTCAGAGGCAGAGAAGGTCGAAATAGTACTTGTGGGCTTACCTCCGGAGTTCAATGCCGTCCTTACCTTAGCATCGTTCTCGTCGGAGCCTCTGCCACTTCAACGTCTCATCGACGTGTTACTCGAGTATGAGAGCCGCTAGGTGCGTGCGGTACAGGACGTGCCTCTTCATGCCAATCTTTTGGAAACTATTCCATCGTCGGCTCTGGTGGGCTCTGTCTGTGGTGGTCGTTCCCCGTCTGAAGGTCGCGGGAGAGGGTTTCGATCCTGCATACAATGTCAGATCTGCGGGTGGTTTGGGCATTTAGCCCAGAGGTGCTTTTTCCGTTATAACCGTGAGTATGATAGCCTATCGTCGGTTGTTCGAACGTCAAATCTGGCGGATCGTCATGGTCCTGGTGTGTCATTTCCGTTTGACAGGACACTGATTTACAAGGCAATGTGGAGGAAGGCAAAAGAGTGCCTTTACGTCGAGCCTGACGTTGGTGGTGGTGGTAGCAAATGCGTTTTTTGGTATCGTAACAAGTTCTAAAGACCGACCAACTACTTGATAGCTAGCCAATGAAGTCGACGTAAAATGTACTTATTAGACTAGTGTTAACTAAGGAAGTTACTAATGTTAGGAAAATGTGCAATAGTTGCCTAGTAGTTTTATGTTTAATATAAGTAGCAATTAAAATATATTTAGTATTCAA**G**TGAGTCTTAGCTTGATTGGTATGGGCATTATCCCCAATGCAGGAAAACTTAGGTTTG

**Supplementary Figure 1 Detailed nucleotide sequence annotation and structural features of the *pGhFAD2-1***

The full-length cloned transcript sequence (1,476 bp) is highlighted in yellow. The precise transcription start site (TSS) and transcription termination site (TTS) are indicated in bold red letters. Sequences sharing homology with the ancestral GhFAD2-1 gene are underlined. The spliced intronic region is characterized by canonical GT-AG splice donor and acceptor sites. The 1,221-bp exogenous insertion, presumably originating from an ectopic genomic region, is denoted in red text.
